# American Medical Association, Section on Stomatology

**Published:** 1902-12

**Authors:** 


### AMERICAN MEDICAL ASSOCIATION, SECTION ON STOMATOLOGY.

(Continued from page 847.)

*Third Session, Wednesday Afternoon, June 11, 1902.*

THE meeting was called to order by the chairman.

A paper entitled "A Comparative Study of the Attachment of the Teeth" was read by Dr. Frederick Noyes, of Chicago.

(For Dr. Noyes's paper, see page 639.)

### DISCUSSION.

*Dr. Eugene S. Talbot.*—The drift of this paper rather confirms that of my own read this morning. The evolution of teeth and their attachment is very similar to that of the dental pulp. In the placoid scale, from which all evolution must start, the fibrous tissue that goes to form the inner part of the tooth has much more nourishment. The pulp keeps up a constant nourishment for the scale throughout the life of the animal. In the second class, where we have the tooth of the shark and the tooth of some of the fossil birds, we have the hinged joint. Where the teeth lie along the borders of the jaws in a fibrous depression we have the highest type of physical development of the teeth. We have the cone-shaped teeth with an immense blood-flow keeping the structures in healthy condition. As we ascend the scale, taking up the third form of teeth attachment, we see changes in the alveolar processes for the purpose of making sockets for the higher tooth development. We have the roots of the teeth developed. The difference in the evolution is very important in the teaching of students.

*Dr. Vida A. Latham.*—I would like to know if true ankylosis has been found in the human? I once had a case which I let Dr. Ford examine, and he thought it as near true ankylosis as he had ever seen, but said he had never known it to occur. I do not say it was overgrowth of the cement, but the jaw was broken in getting the tooth.

The author states that the odontoblasts are first formed on the surface of the pulp. I would like to ask, Why do they form there? There is no more dentine on the surface of the teeth than there is laterally. Another point. Do teeth that are moved in irregularity cases increase in the amount of connective tissue? They may, and that may also cause some teeth to die from circulatory disturbance. I would like to know of some irregularity work and have a chance of extracting teeth to decide this point. We sometimes have fibrous tissue reformed, but usually it lowers the vitality. That would also bear on the different types of teeth.

Dr. E. A. Bogue, of New York City, read a paper entitled "Observations on some Recent Cases of Orthodontia," with illustrations.

(For Dr. Bogue's paper, see page 869.)

DISCUSSION.

*Dr. E. S. Talbot.*—I wish to take exception to the use of the term contraction. The term has crept into the profession without knowing the etiology of the conditions represented in these cases. There is not a contraction. It is an arrested development. Contraction means a condition in which a jaw has reached a certain size and then has grown smaller. Here, and in all of the cases where one jaw is smaller than the other, the term is not a proper one, and it is misleading.

There are two objections to taking hold of irregularities at this particular time. The roots of the permanent teeth are not properly formed, and moving has a tendency to produce deformity of the roots. This has been seen in children who have struck their teeth. The second objection is that when arrest of development takes place we do not know whether that jaw will develop. There may be an arrest of development that never will progress. The arrest may take place in the temporary teeth and not in the permanent. Moving of the teeth is not advisable, because we throw them out of the surface of the alveolar process and there is little or no bone left on the outer border. Such a case is illustrated in Tomes's work on Dental Surgery, where teeth erupted on the outer border of the alveolar process. There is an advantage, on the other hand, in assisting naturally in developing the alveolar processes and the jawbone.

I do not think there is a change in the roof of the mouth as mentioned by the writer, but in the alveolar processes. Some of our neurologic friends in New York have made a great mistake in that point. A paper before one of the societies, I think it was the Odontological Society, spoke of a change in the vault, and there was not a single case reported that shows a particle of change. There was rather an hypertrophy in the alveolar process. This hypertrophy is very common among neurotics. These two children operated upon are certainly what we call degenerate children. I think Dr. Bogue has done a magnificent piece of work, and he has been assisted by the children going abroad. Had they stayed at home the chances are they would not have gotten on so well. This brings up a point that is not mentioned in the text-books,—that all of these children who have arrested development of the jaw are neurotics and degenerates. The presence of the arrest shows that the system is unstable. Whether to commence this work so early or at the period of puberty is a difficult question. I venture to say that if the doctor will watch these children he will find that from twelve to sixteen they will be found very irritable and nervous. Such is my experience in twenty-eight years of practice.

*Dr. Bogue.*—I would like to ask Dr. Talbot to define what he means by "degenerate."

*Dr. Talbot.*—One whose nervous system is unstable and whose physical development is a departure from the normal.

*Dr. Latham.*—The work of painless regulation cases is a very, very strong point. To me these cases are the most objectionable, for the conditions wear upon the nervous and mental system of patients and parents. I was glad to hear the doctor speak of the second and third nut. The little folks will turn the appliances with their tongue back to the old place. If the second nut controls this I am exceedingly glad to know it.

One question from a developmental point. In a vertical section through the face the lower jaw shows a pronounced eruption compared with the upper. I wondered if this made any difference in the eruption of the processes.

*Dr. J. L. Williams,* Boston.—I want to express my approval of this painless operation. My habit has been always to remove the pressure at the first indication of pain. The instrument can be moved intermittently without harm. I have seen mischief resulting when the rubber was not watched. In one case after careful correction of the lower teeth, at the urgent solicitation of the patient's mother and the advice of the former dentist, the regulation of the upper teeth was commenced, with the provision that the patient should come to me for further instructions. This she neglected to do, and though there was much pain, she thought mind was superior to matter, and when I next saw her one of the incisor teeth was dead. The regulation intermittently of teeth is the only safe method.

*Dr. E. A. Bogue* (closing the discussion).—I accept Dr. Talbot's exception very gladly, but will take the liberty of repeating what I did say,—that there was a perceptible change, which lasted temporarily, of the shape of the roof of the mouth. I did not mean that anything but the gum was really affected. In the regulation cases the alternate work and rest is the only way to accomplish results with entire propriety and health. The extraction of teeth to make room is something never as fully understood by me as it is to-day, and I understand it after a pretty severe lesson. I have now in my possession notes of upward of two thousand patients who have passed through my hands, in whose cases I have models taken year after year. The changes that take place before extraction and after extraction are thereby before our eyes. These illustrations of the conditions at varying dates have shown me that the Creator knew a great deal more than we do about how to make room; for the cusping of teeth, upper and lower, with each other is an absolutely essential thing if the mastication of our food is to be performed as nature intended. One of our most noted dental surgeons admitted that the question of occlusion had not been prominent in his mind; that his work had been solely for the appearance of the teeth and not utility. Starting with the principle that the arch generally needs to be extended, and that the cusping of teeth will almost always hold them in place once they have been gotten into place, I inferred that it was much easier to do for a growing child what was required than to wait until that child was more nearly formed, and Dr. Williams's plan has always been operative with me whenever such work has been undertaken with children. I have been very much pleased that these children have never even complained of pain.

Dr. Talbot's definition of degeneracy does not altogether apply to this little chap. I would sometimes address him in German, French, or English, and he would answer with perfect facility. He is as well versed in lessons as a boy of fourteen. I have never seen his superior in intelligence. I acknowledge that he is nervous and that he is delicate. He is a boy who seems to be standing in a precarious condition, and his mother knows it full well. She, too, is one of the brightest women I ever met.

Regarding Dr. Latham's question, I am not able to answer whether the development of the teeth was in the way she mentioned. In these cases I am quite sure that the teeth developed after removal of the obstructions with much greater rapidity than before. What the condition of their roots may be, of course, no one knows.

Dr. G. Lenox Curtis, of New York, read a paper entitled "Electric Ozonation in Neuralgia."

(For Dr. Curtis's paper, see page 635.)

DISCUSSION.

*Dr. J. L. Williams.*—What is the process of the ozone treatment?

*Dr. Curtis.*—I regret exceedingly that the room will not allow me to demonstrate the method; an electric current is necessary. It is applied through a vacuum tube from the street current. The amperage is reduced so that it is perfectly harmless, and the ozone is forced in through the body, so that any internal inflammations are reduced by the action of the electricity and ozone. When I say electricity and ozone I mean electricity, and my definition of electricity is ozone. I have tried to give a definition of electricity, and treated the subject in a paper before the Congress on Tuberculosis, telling how tubercular cases may be cured.

*Dr. Williams.*—Is the apparatus to be seen at any special place?

*Dr. Curtis.*—It is being put on the market.

*Dr. Bogue.*—Is not ozone produced by the atmosphere?

*Dr. Curtis.*—The molecules of ozone in the atmosphere are broken up. It is done by inhalation and by being forced into the tissues. There are great quantities of it given off, so that it is possible to fill a small room sufficiently for a person to sit or lie in it so that it oxidizes the pathogenic conditions in the body through inhalation or by passing through the body. It can be used with both currents, but the same machine will not answer for both.

*Dr. G. F. Eames*, Boston.—I have always understood that ozone is the effect of the electric current upon the oxygen in the atmosphere, and it is difficult to see how this goes through the body, inasmuch as the patient must breathe while it is being applied. I see how, by being taken into the lungs, it can be taken into the blood and through the circulation, but the other way of driving it through the tissues I do not understand; whether it is by the same means as cataphoresis.

*Dr. Curtis.*—My explanation that the ozone does pass into the body lies in the fact that in deep-seated congestions, for instance, congested lung or inflamed liver, when the current is applied over the spot the pain is immediate. In normal tissue there is no pain when electricity is applied. On further treatment the pain subsides and the current passes as through normal tissue. Also, the breath emits the odor of ozone for hours after treatment.

*Dr. Eames.*—The disappearance of pain would not prove to me that this ozone is driven through the tissues, because surface applications will often cause pain to disappear.

*Dr. Curtis.*—I do not think Dr. Eames's question can be answered. A great many scientists, however, accept my reasoning. There is no means of telling at present that the current of electricity is really passed through the body. We know that it traverses the surface. I reason that it does pass through because of these changes.

*Dr. William Knight,* Cincinnati.—We all know the stubborn character of real neuralgia, and that it shows a condition of the nerves which is not normal. Innumerable methods have been adopted in their time, and in special cases are of service to-day. The object of all methods is first to remove the local irritation and then correct the systemic predisposing condition. I would be very loath to believe that pain due to inflammation, to bacterial origin, or pain not neuralgic, is to be suddenly wafted away by something that is called ozone. But, if we have a method which can cure this terrible disease of neuralgia so readily, it is a grand thing, and I would compliment any gentleman who has made such a discovery. Up to the present time the condition has baffled the ingenuity, research, and clinical observation of men in this country and abroad.

*Dr. Curtis* (closing the discussion).—I hope to exhibit the machine to-morrow in the Section on Medicine, where I am to read a paper on "Ozonation upon the Blood," showing improved conditions in the blood. I have been able to get rid of certain pathological conditions present at the beginning of treatment. It will certainly destroy pain, increase nerve-force, improve circulation, and produce assimilation rapidly. I have had cases in which two and one-half grains of morphia were employed daily, and after the first treatment pain ceased and was absent for six months, the treatment being continued throughout the first month every day or every other day. The patient's health so greatly improved that he was able to come to my office a distance of ten miles in a street car, against coming in a carriage and in a very feeble condition. His mental condition, which was badly impaired also, was completely restored. I could cite many similar cases. I have had cases of rheumatism and gout cured apparently as readily as a dose of migraine tablets will relieve pain. The pain and stiffness of the joints entirely disappear under the treatment, so that the patient is restored to usefulness. The remarkable feature is the speedy relief. In some cases in which the patients have been actually sleepless and have suffered from gout for many years pain has entirely disappeared. In other cases weeks may be required to accomplish the same result. Some cases obliged to be carried into the office, in six weeks time have been able to take care of themselves. I think, therefore, that the results of treatment are entirely due to re-establishment of the equilibrium. The red blood-cells are increased, and the white diminished in number. The hæmoblobin is slow to increase. The red cells in chronic cases increase at about one million a month, while the white cells diminish at about eight thousand in the same length of time. In a case of blood-poisoning following abortion the patient lost at the time of the operation some two quarts of blood. There was gonorrhœal infection followed by septic pneumonia. In fourteen days from the first treatment the patient was sitting up, and to-day is perfectly well. All pus disappeared on the fourth or fifth day. The patient was so weak that she could not more than whisper. I saw her the day I left New York, and I have photographs of her blood taken at the beginning of treatment and after. When I began treatment the blood-cell count showed two million five hundred thousand red and sixty-four thousand white cells, the normal being from seven to eight thousand. On the twenty-first day there were three million eight hundred thousand red cells and twenty thousand white cells. The hæmoglobin at that time, which was not noted on the first examination, was sixty per cent. This has increased to eighty and there are five million red cells and eight thousand white cells.

Dr. William Knight, of Cincinnati, Ohio, read a paper entitled "The Modern Dentist from a Medical Stand-Point."

(For Dr. Knight's paper, see page 646.)

DISCUSSION.

*Dr. A. E. Baldwin,* Chicago.—There is little to be said upon this subject more than the essayist has said. I welcome any paper which shows the need of greater breadth in our specialty, and I have been an earnest advocate of medical education of the dental student. I first studied medicine and practised for a number of years, afterwards taking up the specialty of dentistry. I think men are better qualified for the practice of dentistry by such a course of study and preparation. This section has done much to bring dentistry into its proper position, and has also exerted a great influence for good upon the general dental practitioners.

*Dr. M. L. Rhein.*—We all recognize the appropriateness of that which the essayist has presented to us. The need of educating the dentists of the future in the groundwork such as we recognize they should have is an important matter which should occupy our thought. This groundwork should be as thorough as for any department in the broad field of medicine. It is as difficult to differentiate between the normal and pathologic conditions of the mouth as it is those conditions in any other part of the body.

*Dr. E. A. Bogue.*—In the matter of reflex inflammations and of dental difficulties being at the base of ocular and oral troubles, it so happened in the cases referred to by Dr. Knight that there had been serious neglect. Here we as dental specialists can render valuable aid. While agreeing with all that has been said regarding curative methods, I feel that there are sometimes other methods to be adopted.

*Dr. Eames.*—I am grateful for the paper that has been read. It perhaps does not call so much for discussion as for words of approval, making emphatic the one grand principle we are all working for. It seems to me that such papers should be read before other sections of this Association.

*Dr. Knight* (closing the discussion).—I do not know that I have anything further to state. The object of the paper was to turn the attention of the medical and dental professions to the fact that there should be more co-operation between the two. Many practitioners are largely astray in their knowledge of dental affections. They need to be better informed as to when they shall direct a patient to a dentist. If we can get the oculist to more generally appreciate the connection of eye affections with the fifth cranial nerve, and if the dental student has impressed upon him the necessity for a practical knowledge of anatomy and physiology, much advance will be made in the relief of suffering. There is a tendency for specialism to become narrow. Adenoids have been taken out indiscriminately, and there has been hypertrophy of the alveolar process. In some cases operation is permissible, but in the majority of cases general treatment is sufficient to guard against the condition. Cicatricial tissue and secondary contraction take place after operation, and these may cause more inconvenience than the primary condition. I think the laryngologist should co-operate more with the general practitioner. Only a general practitioner knows how to treat many cases that go to the oculist. I think the time is coming when this general recognition will be shown to the dental surgeon.

Dr. G. F. Eames, of Boston, read a paper entitled "Oral Hygiene."

(For Dr. Eames's paper, see page 881.)

DISCUSSION.

*Dr. Vida A. Latham.*—I think all of us are familiar with Dr. William Hunter's paper on "Oral Sepsis," a paper which has done more towards uniting the medical and dental professions than any other paper ever written in the history of medicine. It is quoted all over the world, showing the great importance of oral sepsis. I would also like to call attention to that queer disease known as pernicious anæmia. There we get a most peculiar glossitis, a condition that is so characteristic as to be almost one of the diagnostic points in pernicious anæmia. We can get from the lymphatics of the tongue almost pure cultures of the streptococcus longus.

*Dr. Talbot.*—The essayist says that a perfectly healthy mouth will not emit odor, and speaks of decomposition of food about the teeth producing foul odors, etc. I do not entirely agree with those statements. The amount of food which collects about the roots of the teeth is very small, indeed, and I question the amount of odor therefrom. Again, the mouth may be healthy, but there may be odor from autointoxication due to indigestion or to the secretions locked up in the system. In cases of interstitial gingivitis or alveolar pyorrhœa we have the worst forms of odor. In a great many teeth which I have cracked open the odor was frightful, and yet the pulps were still alive. In cases of extreme absorption of the alveolar process with exposed tubuli the odor is also bad.

In the treatment, I must take exception to the use of a soft or wet brush. My experience is that there is not a brush made that is sufficiently stiff for my patients. In order to have a healthy mouth we must have a gum massage brush. I am glad to hear the protest regarding the examination of the mouths of school children. I think the habit of examining the mouths of these children without thorough cleansing of the fingers is abominable.

*Dr. M. L. Rhein.*—Dr. Eames speaks of three minutes being advised for brushing the teeth. I insist that my patients shall give from four to five minutes to the brushing. I think the average patient will give about thirty seconds to this important matter, and I am accustomed to lay down a law to be rigorously observed. I agree with Dr. Talbot that the bristles of a brush cannot be too hard. I instruct my patients to use the flat sides on the gums as near the roots as possible in order to stimulate the capillaries of the gum tissue. Recession of the gums or necrosis of the tissues can be greatly improved if the tone of the tissues is kept up by proper massage. This point I especially emphasize in my instructions to assistants.

*Dr. G. V. I. Brown,* Milwaukee.—We all have our own ways of accomplishing the same results. The broader view of the paper is its significance in relation to the prophylactic feature. I want to correct what may appear in the print to be, as I am sure it was not intended, a protest against the examination of school children. Very little has grown out of these examinations, yet there is a work which ought to be looked after. I think many of the ills of their future lives could be avoided by proper attention to this examination. Matters pertaining to nose- and mouth-breathing and the general disinfection of the mouth are of the greatest importance. Much could be done in checking epidemics of diphtheria and of tuberculosis by insisting that a large vessel of water be placed at the door of the school-room and the children made to take a mouthful of water and hold it in their mouths for a few minutes. Whatever may be done with the sputum, the destruction of bacilli before they get into the air would be true prophylaxis.

*Dr. A. E. Baldwin.*—I believe practical results are to be obtained by anything that will properly cleanse the system and promote a healthy condition of the mouth. It seems to me it is hard to determine whether odors come from within or beyond the mouth. I think the fears regarding tuberculosis are carried to the extreme. I thoroughly believe that tuberculosis is a great scourge, and that all possible should be done to prevent it, but when it is realized that during the life of the human race it is estimated by careful investigators that fully seventy per cent. of the people who attain adult life have sometimes during their life tubercular trouble and get well, the disease does not seem so hopeless as might at first be considered. I think care in the disposition of the sputa will bring the best results in preventing the spread of the disease. I am a thorough believer in cleanliness in any way it can be accomplished.

*Dr. Eames* (closing the discussion).—In regard to the healthy mouth not emitting a foul odor, I will admit that the mouth may be the outlet of an odor that may come from a distant part, but that distant part being seriously diseased, it is a question in my mind whether the mouth can still remain in a perfectly healthy condition. When I made that statement I considered it carefully.

The brush I leave to the discretion of the dentist. I said, dried, the brushes are sufficiently stiff. The idea was to make a distinction between the wet and the dry brush. I believe we can cleanse the teeth better with the wet brush if the bristles are sufficiently stiff. I see no harm in the use of the finger over the gums and it may help the puffy condition of gums as I have seen them. I practically leave the time for brushing the teeth to the patient. I say brush them thoroughly, and when you think you have done this, brush them one minute longer. I was careful, in making my reference to the school children's examinations, not to state how these were made. I think you would infer that they were not antiseptic. I did make emphatic the fact of the rights of parents of poor children in having the decision of who shall make these examinations.

Adjourned to Thursday, June 12, at two P.M.

(To be continued.)

